# Safety and effectiveness of vortioxetine for major depressive disorder: Real-world evidence from a population-based study in South Korea

**DOI:** 10.3389/fpsyt.2023.1075939

**Published:** 2023-03-02

**Authors:** Seok Woo Moon, Jee Wook Kim, Do Hoon Kim, Kyu Young Lee, Elin Heldbo Reines, Minah Lee, Yoo Jin Park

**Affiliations:** ^1^Department of Neuropsychiatry, Konkuk University School of Medicine, Chungju-si, Republic of Korea; ^2^Department of Neuropsychiatry, Hallym University Dongtan Sacred Heart Hospital, Hwaseong-si, Republic of Korea; ^3^Department of Psychiatry, Hallym University Chuncheon Sacred Heart Hospital, Chuncheon-si, Republic of Korea; ^4^Mind-Neuromodulation Laboratory, College of Medicine, Hallym University, Chuncheon-si, Republic of Korea; ^5^Department of Psychiatry, Nowon Eulji Medical Center, Seoul, Republic of Korea; ^6^Department of MSC Vortioxetine and Established Products, H. Lundbeck A/S, Copenhagen, Denmark; ^7^Department of RA&MA, Lundbeck Korea Co., Ltd., Seoul, Republic of Korea

**Keywords:** antidepressants, major depressive disorder, non-interventional, post-marketing surveillance, South Korea, vortioxetine

## Abstract

**Background:**

A post-marketing surveillance study was conducted to assess the real-world safety and effectiveness of vortioxetine for the treatment of major depressive disorder (MDD) in South Korea.

**Methods:**

Adult patients aged 19–94 years receiving vortioxetine for MDD at 72 hospitals and clinics in South Korea between 19^th^ August 2014 and 18^th^ August 2020 were included. Patients were followed for up to 24±2 weeks, at up to three visits. Adverse events (AEs) and effectiveness, assessed by both clinician and patient-reported measures, were analyzed.

**Results:**

A total of 3,263 patients (mean age: 51.28 years) were included in the safety set; 1,095 were aged ≥65 years. The majority of the safety set (61.97%) were female. The overall rate of any AEs and serious AEs were 17.13 and 1.56%, respectively. The majority of AEs were mild (88.32%). The rates of AEs did not differ statistically by age (≥65 years: 16.89% [185/1,095] versus <65 years: 17.25% [374/2,168)], *p*=0.7989), sex (male: 15.95% [198/1,241] versus female: 17.85% [361/2,022], *p*=0.1623), or liver impairment (with liver impairment: 20.90% [14/67] versus without liver impairment: 17.05% [545/3,196], *p*=0.4087). Effectiveness was assessed in 1,918 patients. By 24±2 weeks, there were significant clinical improvements from baseline, assessed by change in Montgomery-Asberg Depression Rating Scale total score (mean±standard deviation [SD]: -10.49±9.42 points, *p* <0.0001), the proportion of patients with improved symptoms using the Clinical Global Impression - Improvement scores (79.29%), and in both patient-reported measures, with a significant improvement in the Korean Version of the Perceived Deficits Questionnaire-Depression (mean±SD: -6.06±13.23, *p* <0.0001) and Digit Symbol Substitution Test (mean±SD: 4.83±9.81, *p* <0.0001) total scores from baseline. Similar to the safety profiles, the proportions of patients with improved symptoms compared with baseline using the Clinical Global Impression – Improvement scores did not differ by age (≥65 years: 82.09% versus <65 years: 78.32%, *p*=0.0511), sex (male: 77.45% versus female: 81.01%, *p*=0.0587), or liver impairment (with liver impairment: 67.57% versus without liver impairment: 79.85%, *p*=0.0663).

**Conclusion:**

Vortioxetine appears to be well-tolerated and effective for treating MDD patients in the real-world setting in South Korea, irrespective of age, sex, and liver impairment, reflecting the known profile of vortioxetine based on studies worldwide.

## 1. Introduction

Major depressive disorder (MDD) is one of the most common psychiatric disorders, with an approximate global prevalence of 3,440 per 100,000 (1). It is associated with reduced productivity and increased risk of morbidity and suicide (2, 3), which contribute to it having been identified as one of the 10 most important drivers of global disease burden among people aged 10–49 years (4). Furthermore, even when patients receive treatment for MDD, they can remain at risk of relapse (5). The burden of MDD has been rising rapidly worldwide, with the age-standardized incidence increasing by 50% from 1990 to 2017 (6). Among 195 countries and regions, the increase in age-standardized incidence rate in South Korea was third largest following Belgium and Guyana (6). In addition, South Korea was reported to have the highest suicide rate between 2003 and 2019 among countries listed by the Organization for Economic Co-operation and Development (7). Therefore, MDD poses substantial burden on patients, the healthcare system and society, highlighting the need for effective treatments for this condition worldwide and in South Korea.

Several classes of antidepressants are reimbursed for the treatment of MDD in South Korea, including selective serotonin reuptake inhibitors (SSRIs), tricyclic antidepressants (TCAs), serotonin-norepinephrine reuptake inhibitors (SNRIs), serotonin antagonist and reuptake inhibitors, and noradrenergic and specific serotonergic antidepressants (5). While these antidepressants are available for MDD, a substantial proportion of MDD patients may exhibit suboptimal responses for a variety of reasons (8). These may be attributable to several patient-, clinician- or treatment-related factors, including presence of comorbidities, non-adherence to antidepressant treatment or inadequate dose and/or duration of treatment (9, 10). For example, the presence of psychiatric comorbidities has been associated with poor outcomes in depression (9). This may be linked with issues such as non-adherence, which is common in chronic psychiatric conditions (9). Additionally, patients with common comorbidities such as cardiovascular diseases or diabetes mellitus have been shown to be at higher risk of depression, with several classes of drug (e.g., beta-blockers, corticosteroids) even capable of causing depression (11), highlighting the importance of achieving remission in those with comorbidities. Treatment- or physician-related factors such as dosage/duration of treatment are also difficult to determine in the real-world, with standard recommendations for dosage and duration being based on clinical trials, with refractory patients in the real-world often requiring higher dosages or exhibiting slower responses (9). Suboptimal response may also be related to the mechanism of action of the antidepressants themselves. SSRIs and SNRIs target the monoaminergic system by blocking monoamine reuptake, however the exact mechanism of action remain unknown and its effect can be insufficient for patients to achieve remission (12).

Vortioxetine is a novel antidepressant, differing from SSRIs with its multimodal mechanism of action. It displays high affinity for the serotonin transporter, thus selectively blocking serotonin reuptake as well as directly modulating pre- and post-synaptic 5-HT receptor activity (including agonism of the 5-HT_1*A*_ and 5-HT_1*B*_ receptors and antagonism of the 5-HT_3_, 5-HT_1*D*_, and 5-HT_7_ receptors) (13–15). It was approved by the Ministry of Food and Drug Safety (MFDS) for the treatment of MDD in South Korea in 2014 and in 2021 was recommended for first-line use by the Korean Medication Algorithm for Depressive Disorder 2021 (16, 17).

Globally, the safety and efficacy of vortioxetine for the treatment of patients with MDD, including those with inadequate response to SSRI or SNRI monotherapy, have been demonstrated in several randomized, placebo-controlled, 6 or 8-week clinical trials in patients with MDD (18, 19). Vortioxetine has also been found to be effective in specific patient subgroups, including those with comorbidities such as MDD and anxiety disorder (20), comorbid cardiovascular diseases or diabetes mellitus (21), or patients aged ≥65 years (22). These findings have been reflected in the real-world, in settings ranging from Europe to South East Asia and Taiwan (23–26).

Following its approval in 2014, as per MFDS requirements (27), a post-marketing surveillance (PMS) study was set up with the aim of assessing the safety and effectiveness of vortioxetine over a 6-year period, from 2014 to 2020 in MDD patients in the real-world, non-interventional setting, as per local prescribing information in South Korea (17, 27).

## 2. Materials and methods

### 2.1. Study design

This was a prospective, multicenter, non-interventional, non-comparative PMS cohort study in South Korea. The study was conducted in 72 hospitals and clinics with mental health and/or neuropsychiatric departments that prescribed vortioxetine for treatment of MDD, with data collection commencing on 13^th^ June 2016 and ending on 14^th^ August 2020.

Vortioxetine was prescribed independently of the study and according to the local prescribing information; the starting doses of vortioxetine in adults (aged 19–64 years) and elderly patients (aged ≥65 years) were 10 mg and 5 mg once daily, respectively. In adults, the dose could be modified from 5 to 20 mg, depending on treatment response. Reasons for dose modification and/or discontinuations during the course of the study were recorded. As patients were assessed in the real-world, non-interventional setting, patients could also receive other concomitant medications during the study period at the physicians’ discretion, including antidepressants administered 1 month prior to participation and/or during the study period, irrespective of administration status of vortioxetine, as long as the prescription aligned with local prescribing information. Types of commonly prescribed concomitant antidepressants included escitalopram and trazodone; types of commonly prescribed concomitant medications included choline alfoscerate, lorazepam and clonazepam. Patients who discontinued vortioxetine could receive other appropriate treatment. The study results were not affected by the receipt of concomitant medications or antidepressants during the study period.

Patients were followed for up to 24±2 weeks at up to three visits, with data collected at each visit *via* electronic case report form (eCRF). The first, baseline visit (Visit 1, week 0) collected informed consent and baseline characteristics. At the short-term visit; Visit 2 (8±2 weeks after Visit 1, mandatory) and the long-term visit; Visit 3 (24±2 weeks, optional), patient characteristics and assessments of safety and effectiveness were conducted. Additional visits beyond Visit 2 were arranged at the investigators’ discretion.

The study was conducted in alignment with the regulations of MFDS (MFDS notification number 2014-61) and was approved by Institutional Review Boards (IRBs) at each study site or a centralized IRB, designated by the Ministry of Health and Welfare when a study site did not have its own IRB. The IRB approval number of the first individual site was PMS2016-010-021. Informed consent was provided by patients prior to registering for the study.

### 2.2. Participants

Patients who provided informed consent prior to enrollment, were naïve to vortioxetine for the treatment of MDD, and received at least one administration of vortioxetine based on eCRF records during the study duration were eligible. Patients who were deemed as hypersensitive to any ingredient of the drug and/or had a history of monoamine oxidase inhibitors within 14 days prior to enrollment were excluded. Vortioxetine is not approved for the treatment of MDD in pediatric and adolescent patients, and thus patients aged <19 years were not enrolled in the study.

### 2.3. Study procedures and evaluations

#### 2.3.1. Baseline characteristics

General demographics, presence of comorbidities, duration of MDD episodes, concomitant medication usage, and the dosage, duration, and reason for initiating vortioxetine were assessed at baseline visit.

#### 2.3.2. Safety

All AEs spontaneously reported by the patient or observed by the investigator were recorded in e-CRFs from the beginning of the study. Serious AEs were reported to the study sponsor and relevant authorities according to local regulations. The AEs were assessed and recorded using World Health Organization Adverse Reactions Terminology (WHOART). All AEs were graded by severity (mild, moderate, and severe) and system-organ class (SOC; based on WHOART). The attribution of AEs to vortioxetine was assessed by investigators as: certain; probably/likely; possible; unlikely; conditional/unclassified; not assessable/unclassifiable. Patients with non-serious AEs were followed up until AEs were resolved or stabilized; those with serious AEs (SAEs) were followed up until resolved. Outcomes of AEs were categorized as: recovered; recovered with sequelae; not recovered; recovering; fatal. Actions taken to AEs were classified as: permanent discontinuation; temporary discontinuation; dose reduction; dose increase; no change.

#### 2.3.3. Effectiveness

The Montgomery-Asberg Depression Rating Scale (MADRS) was used to assess the severity of depressive symptoms (28). This consists of 10 items designed to assess symptoms of depression on a seven-point scale, ranging from 0 indicating the absence of symptoms to 6 indicating severe symptoms. The total score for the 10 items therefore ranges from 0 to 60 and the change from baseline in this score was used to assess the improvements in depressive symptoms. Treatment response was defined as a ≥50% reduction in MADRS total score at Visit 2 or Visit 3 compared to baseline score. Remission was defined as MADRS score ≤10 at Visit 2 or Visit 3.

The Clinical Global Impression-Severity of Illness (CGI-S) and Clinical Global Impression-Improvement (CGI-I) tools were used to assess the clinical characteristics of MDD patients. CGI-S is a seven-point scale that assesses a patient’s severity of symptoms related to mental health, with a higher score indicating a more severe illness (29, 30). CGI-S was used to assess the severity of MDD at baseline. CGI-I is a seven-point scale that allows investigators to assess the degree of change in a patient’s symptoms and ranges from 1, being very much improved to 7, being very much worse (30). Short- and long-term effectiveness of vortioxetine were defined as the proportions of patients that showed improvements in CGI-I scores from baseline at Visits 2 and 3. To provide a single summary measure of effectiveness, the overall effectiveness was analyzed based on CGI-I scores at Visit 2 for patients with short-term vortioxetine treatment (<22 weeks) or at Visit 3 for patients with long-term treatment (≥22 weeks). The rate of effectiveness was calculated by classifying “Improved” categories as “effective,” and categories under “No change” and “Worse” as “ineffective.”

Patient-reported cognitive impairment was assessed at baseline, Visit 2, and Visit 3 using the Korean Version of the Perceived Deficits Questionnaire-Depression (PDQ-K) and the Digit Symbol Substitution Test (DSST). The PDQ-K consists of 20 items that assess cognitive dysfunction in patients with depression (31). The score of each item can range from 0 to 4, with a higher score indicating more severe cognitive dysfunction. The total score for the 20 items can range from 0 to 80. DSST assesses patients’ cognitive dysfunction related to performing everyday tasks, using pairs of symbols and numbers (32). The score can range from 0 to 133, with a higher score indicating better cognitive function.

#### 2.3.4. Sample size

The sample size was determined in alignment with the requirement for a PMS of a new antidepressant by the MFDS, which states that a new drug should be re-examined in at least 3,000 patients. The safety and effectiveness of the drug at the optional long-term follow-up at Visit 3 (24±2 weeks) were assessed in at least 10% of the MDD patients included in the study, as per MDFS requirements.

### 2.4. Statistical analysis

Descriptive statistics pertaining to each patient’s baseline characteristics and data on safety and effectiveness were analyzed and presented. Patients in the safety assessment consisted of those who had been evaluated for safety through follow-up observation after administration of vortioxetine at least once according to the label. The effectiveness assessment patients consisted of all patients who were included in the safety analysis set, administered vortioxetine at least once, and had at least one CGI-I score available at either Visit 2 or Visit 3. Mean, standard deviation (SD), median, and ranges were derived for continuous data and frequencies and percentages were derived for categorical data. In order to evaluate the impact of key demographic and clinical characteristics on the safety and effectiveness of vortioxetine, the incidence of AEs and the rate of improvements in mental health-associated symptoms, assessed using CGI-I scores at Visit 2 or Visit 3, were assessed by sex, by age group (<65 years or ≥65 years), and in patients with or without liver impairment. Chi-squared tests or Fisher’s exact tests were used to compare outcomes between patients in these subgroups. Paired *t*-tests were used to compare outcomes at baseline, Visit 2 and Visit 3. A *p*-value <0.05 was considered statistically significant. Confounding factors were not adjusted for in the subgroup analyses. All statistical analyses were carried out using the SAS Software version 9.4 (SAS Institute, North Carolina, USA).

## 3. Results

### 3.1. Patient disposition and baseline characteristics

**FIGURE 1:**
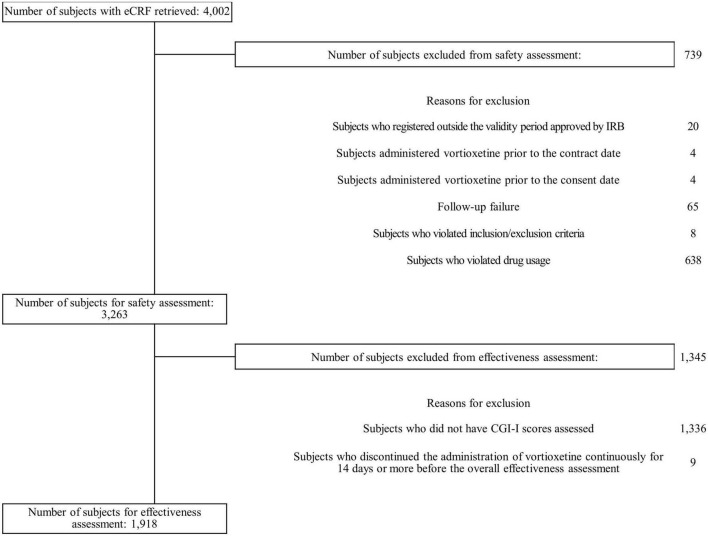
Patient flow diagram. CGI-I, Clinical Global Impression-Improvement; eCRF, electronic case report form; IRB, institutional review board.

Of 4,002 patients’ e-CRFs, 739 were excluded from the safety assessment, the majority due to violation of the drug dosage stated in the local prescribing information (*n*=638) (Figure 1). Of 3,263 in the safety population, 1,918 were included for the effectiveness assessment as 1,336 patients did not have CGI-I scores assessed and the remaining 9 patients discontinued vortioxetine.

As shown in Table 1, the mean age of the safety population was 51.28±20.42 years and 33.56% of the population were aged ≥65 years. The majority of the safety population were female (61.97%). None of the female patients were pregnant at baseline or became pregnant during the follow-up. The majority of the population (54.83%) had a history of medical conditions besides MDD. 87.62% of the population were on concomitant medications, of which 60.48% were on concomitant antidepressants. CGI-S was collected in all patients at baseline, however analysis of CGI-S scores was conducted only in the 1,918 patients included in the effectiveness population; among them, 22.94% had borderline mental illness or were mildly ill.

**TABLE 1:** Baseline patient characteristics of the safety population.

Patient characteristics	Safety population	*N*
Age, years, mean (SD) Aged ≥65 years), *n* (%)	51.28±20.42 1,095 (33.56)	3,263
Female, *n* (%)	2,022 (61.97)	3,263
Pregnant, *n* (%)	0	2,022
Mean weight, mean kg (SD)	62.22±13.03	2,271
Presence of other medical history, *n* (%) Renal impairment Liver impairment	1,789 (54.83) 46 (1.41) 67 (2.05)	3,263
Severity of MDD using CGI-S Normal Borderline Mild Moderate Markedly ill Severe Extreme	0 (0.00) 48 (2.50) 392 (20.44) 781 (40.72) 469 (24.45) 217 (11.31) 11 (0.57)	1,918
Duration of MDD^, days, mean (SD)	766.28±1,541.40	2,527
Duration of MDD episode, days, mean (SD)	317.18±727.21	1,468
Reason for initiating vortioxetine, *n* (%) Treatment-naïve Ineffective previous antidepressants AEs of previous antidepressants Lack of compliance Others	1,605 (49.19) 1,568 (48.05) 55 (1.69) 27(0.83) 8(0.25)	3,263
Duration of vortioxetine use, days, mean (SD)	81.84±62.36	2,653
Long-term use (≥154 days/22 weeks)	677(25.49)	2,656
Starting dose in adults aged 19–64 years 10 mg/day 5 mg/day	1,824 (84.13) 344 (15.87)	2,168
Daily dose, mg/day, mean (SD)	9.52±3.56	2,637
Concomitant medications*, *n* (%)	2,859 (87.62)	3,263
Concomitant antidepressants, *n* (%)	1,729 (60.48)	2,859

^Duration of MDD was calculated as follows: Duration of MDD=[First administration start date] – [Initial Diagnosis Date of MDD] + 1; *Any medications besides major depressive disorder treatments during the study period or major depressive disorder treatment drugs within 1 month before study participation and/or during the study period. AE, adverse event; CGI-S, Clinical Global Impression-Severity of Illness; MDD, major depressive disorder; SD, standard deviation.

Approximately half (49.19%) were treatment-naïve patients who initiated vortioxetine as the first antidepressant regimen, while 48.05% initiated the drug because previous antidepressants were ineffective. In the majority of patients aged 19–64 years (84.13%), the starting dose was 10 mg/day and 25.49% received long-term vortioxetine treatment, defined as ≥22 weeks.

### 3.2. Safety

The overall rate of any AE was 17.13% (745 events in 559 patients) (Table 2) and the majority of these (88.32%; 658/745 events) were classified as mild. Most AEs were unlikely (39.87%; 297/745 events) or possibly (46.31%; 345/745 events) attributed to vortioxetine, with a small proportion being certainly (2.15%; 16/745 events) or likely (9.53%; 71/745 events) attributed. The majority of AEs were classified as recovered or were recovering at the time of assessment (88.19%; 657/745 events), 10.34% (77/745 events) had not recovered, and 0.94% (7/745 events) had recovered with sequelae. Discontinuation due to AEs occurred in 29.40% (219/745 events) (Table 3).

**TABLE 2:** Summary of adverse events (AEs).

Types of AEs	Number of patients (%) (*N*=3,263)	95% CI	Number of events
Any AEs	559 (17.13)	15.85–18.47	745
Serious AEs	51 (1.56)	1.17–2.05	62

AE, adverse event.

**TABLE 3:** Action taken to AEs.

Types of action	Number of events (%) (*N*=745)
No change	438 (58.79)
Permanent discontinuation	219 (29.40)
Temporary discontinuation	8 (1.07)
Dose reduction	71 (9.53)
Dose increase	9 (1.21)

AE, adverse event.

When AEs were classified by SOC, the SOC with most frequent AEs were: gastro-intestinal (GI) system disorders occurring in 8.31% of patients (271/3,263; 307 events), followed by psychiatric disorders in 3.22% (105/3,263; 113 events) and central and peripheral nervous system disorders in 3.16% (103/3,263; 110 events) of patients. The most frequent type of AEs, by preferred term (PT) were: nausea occurring in 5.12% of patients (167/3,263; 169 events), followed by dizziness in 1.32% (43/3,263; 43 events) and headache in 1.23% (40/3,263; 40 events) of patients. The rate of sexual dysfunction (male: 0.06%; 2/3,263, female: not available) was low. In total, 62 SAEs were reported in 51 patients (1.56%; 62 events). Classifying the SAEs by PT, the most frequent were abdominal pain, depression (aggravated) and intentional self-injury.

**FIGURE 2:**
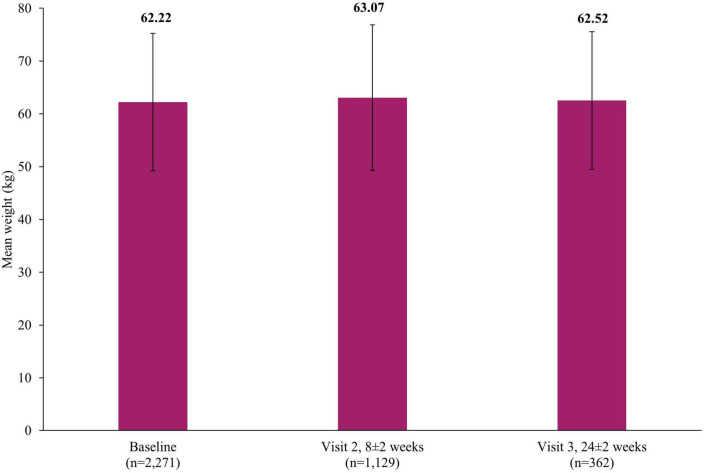
Mean weight at baseline, Visit 2 and Visit 3. Error bars represent standard deviations.

The rates of weight increase (0.21%; 7/3,263; 7 events) and weight decrease (0.06%; 2/3,263; 2 events) as AEs were low. Figure 2 presents the mean weight at baseline (62.22±13.03 kg), Visit 2 (63.07±13.78 kg, 8±2 weeks) and Visit 3 (62.52±13.03 kg, 24±2 weeks).

Of the total safety population, 1,095 patients were aged ≥65 years. The unadjusted rates of AEs in those aged ≥65 years (16.89%; 185/1,095; 259 events) and patients aged <65 years (17.25%; 374/2,168; 486 events) were not significantly different (*p*=0.7989). The unadjusted rates of AEs between male (15.95%; 198/1,241; 267 events) and female (17.85%; 361/2,022; 478 events) patients were not significantly different (*p*=0.1623). Of the total safety population, 67 patients had liver impairment; the unadjusted rates of AEs in these patients with liver impairment (20.90%; 14/67; 20 events) and those without liver impairment (17.05%; 545/3,196; 725 events) were not significantly different (*p*=0.4087). The type and overall profile of reported AEs were not associated with new safety concerns.

### 3.3. Effectiveness

**FIGURE 3:**
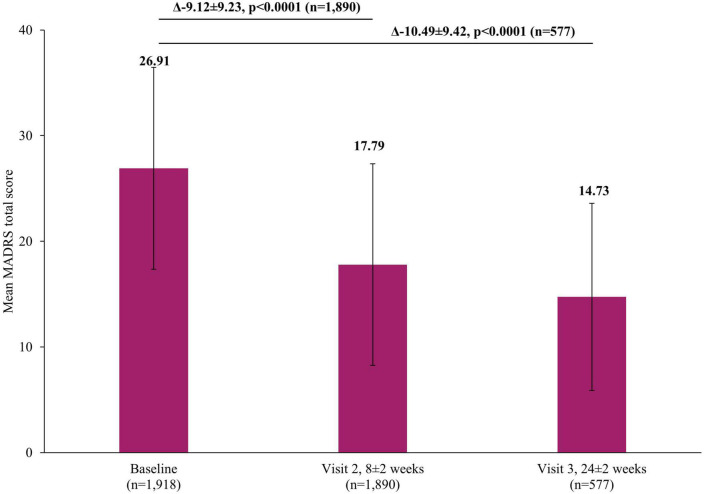
Change in MADRS total score from baseline. The mean MADRS total score at Visit 1 was based on all patients, not just those that also had data at Visit 2 or Visit 3, however, when analyzing the difference between the visits (Visit 1 to Visit 2 and Visit 1 to Visit 3), scores of patients who had scores at both visits were included. Therefore paired *t*-tests assessed the difference at Visit 2 (8±2 week) versus Visit 1 (0 week) in 1,890 patients and difference at Visit 3 (24±2 week) versus Visit 1 (0 week) in 577 patients. Error bars represent standard deviations. MADRS, Montgomery-Asberg Depression Rating Scale.

There was a significant reduction in the severity of depressive symptoms after treatment with vortioxetine, based on the mean MADRS total score which decreased from baseline by 9.12±9.23 points at the short-term Visit 2 (8±2 weeks) and 10.49±9.42 points (*p* <0.0001) at long-term Visit 3 (24±2 weeks) (Figure 3). This improvement in severity was reflected in the rates of treatment response (defined as patients with a ≥50% reduction in MADRS total score at Visit 2 or Visit 3 compared to baseline score) which increased from 31.48% (95% confidence interval [CI]: 29.39–33.63) at Visit 2 to 43.67% (95% CI: 39.58–47.83) by Visit 3. Similarly, the rate of remission (defined as patients with MADRS score ≤10 at Visit 2 or Visit 3), at Visit 2 was 25.40% (95% CI: 23.45–27.42) which increased to 35.88% (95% CI: 31.96–39.94) by Visit 3.

**FIGURE 4:**
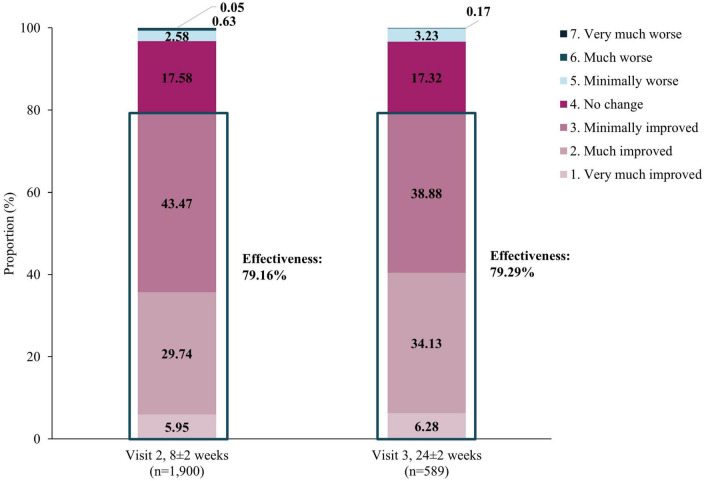
Effectiveness using CGI-I assessment. Effectiveness of vortioxetine was defined as the proportion of patients that showed improvements in CGI-I scores from baseline at Visits 2 (8±2 weeks) and 3 (24±2 weeks). Effectiveness was calculated by classifying “Improved” categories as “effective”, and categories under “No change” and “Worse” as “ineffective”. CGI-I, Clinical Global Impression-Improvement.

Improvements in mental health-associated symptoms were also observed *via* CGI-I scores. Overall, the rates of improvement were comparable at both visits. By Visit 2, improvement was observed in 79.16% patients and this was maintained at Visit 3, with 79.29% patients improved (Figure 4). The overall effectiveness of vortioxetine (at Visit 2 or 3 by CGI-I score) was therefore 79.61%.

Of 1,918 patients included in the effectiveness assessment, 659 were aged ≥65 years. Overall the unadjusted effectiveness measured by CGI-I in those aged ≥65 years was 82.09% which was not significantly different to the effectiveness rate in those aged <65 years (78.32%, *p*=0.0511). There was also no significant difference in unadjusted effectiveness by sex (male: 77.45%; female: 81.01%, *p*=0.0587) or between those with liver impairment and without liver impairment (67.57% versus 79.85%, *p*=0.0663).

**FIGURE 5:**
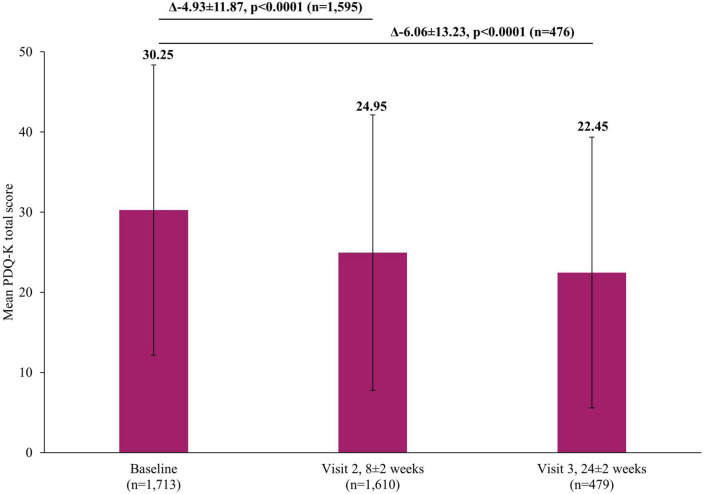
Change in PDQ-K total score from baseline. The mean PDQ-K total score at Visit 1 was based on all patients, not just those that also had data at Visit 2 or Visit 3, however, when analyzing the difference between the visits (Visit 1 to Visit 2 and Visit 1 to Visit 3), scores of patients who had scores at both visits were included. Therefore paired *t*-test assessed the difference at Visit 2 (8±2 week) versus Visit 1 (0 week) in 1,595 patients and difference at Visit 3 (24±2 week) versus Visit 1 (0 week) in 476 patients. Error bars represent standard deviations. PDQ-K, Korean version of Perceived Deficits Questionnaire-Depression.

**FIGURE 6:**
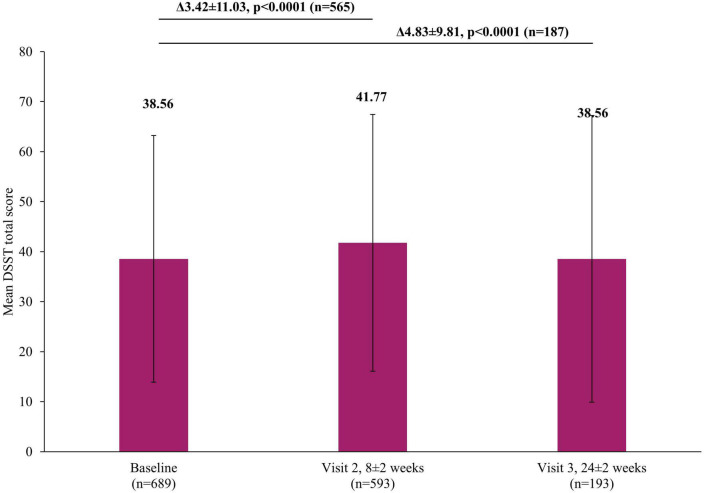
Change in DSST total score from baseline. The mean DSST total score at Visit 1 was based on all patients, not just those that also had data at Visit 2 or Visit 3, however, when analyzing the difference between the visits (Visit 1 to Visit 2 and Visit 1 to Visit 3), scores of patients who had scores at both visits were included. Therefore paired *t*-test assessed the difference at Visit 2 (8±2 week) versus Visit 1 (0 week) in 565 patients or difference at Visit 3 (24±2 week) versus Visit 1 (0 week) in 187 patients. Error bars represent standard deviations. DSST, Digit Symbol Substitution Test.

In addition to the significant improvements on clinical scales assessing depression severity, significant improvements were also reported by patients themselves in both the short and long-term. A significant reduction in self-perceived deficits using PDQ-K was observed, with the mean score decreasing by 4.93±11.87 points (*p* <0.0001) at Visit 2 and by 6.06±13.23 (*p* <0.0001) at Visit 3, compared to baseline (Figure 5). Similarly, there was a significant improvement in patient-reported cognitive function using the DSST, with the mean score increasing by 3.42±11.03 points (*p* <0.0001) at Visit 2 and by 4.83±9.81 (*p* <0.0001) at Visit 3, compared to baseline (Figure 6).

## 4. Discussion

This study demonstrated that vortioxetine was long-term safe and effective in MDD patients in South Korea with a wide range of clinical and sociodemographic characteristics.

The overall rate of AEs was 17.13%, with the most frequent types being nausea (5.12%), dizziness (1.32%) and headache (1.23%). The rate of overall AEs as well as that of frequent AEs was lower than observed in RCTs (overall AEs: 65.3%, nausea: 23.6%, dizziness: 5.6%, headache: 12.7%) (21), however, this is more comparable to rates observed in other real-world studies of vortioxetine in Western (RELIEVE: overall AEs of 21.2%, nausea of 8.2%, headache of 1.5% over 6 months) or Asian settings (TREVIDA: overall AEs of 6.7%, nausea of 1.2%, dizziness of 0.8% over 3 months; RELIEVE China: overall AEs of 42.1%, nausea of 18.3%, dizziness of 3.2%, headache of 1.3% over 6 months) (23, 25, 33). These differences may be attributable to differences between real-world and experimental clinical settings, such as more heterogeneous patient populations and less frequent follow-up visits in the real-world, leading to lower rates of AEs (34). Overall, the tolerability profile of vortioxetine was consistent with its known profile in the real-world setting (23, 25, 33).

In addition to the reassuring safety profile, the overall effectiveness of vortioxetine measured by CGI-I was shown to be approximately 80% at both Visits 2 and 3. Its effectiveness was also reflected in the significant decrease in clinical symptoms *via* MADRS total scores achieved at Visits 2 (9.12±9.23) and 3 (10.49±9.42). Similarly, patients reported significant improvements in cognitive function *via* both DSST and PDQ-K. These results echoed the significant improvements in clinical symptoms and patient reported cognitive function reported in RCTs and/or real-world studies of vortioxetine (19, 25, 26, 33, 35, 36).

When reviewing the safety and effectiveness data in key subgroups, a similar profile was observed, irrespective of age, sex, or liver impairment, confirming the usefulness of vortioxetine in real-world patients. Of particular note was the observation of no statistically significant difference in AEs between patients aged ≥65 years and <65 years. Given that older patients often present with more comorbidities than younger patients, this has previously been reported to lead to higher rates of AEs in older patients (37, 38). Despite these previous findings, the current study suggests that vortioxetine presents a treatment option for older MDD patients that balances safety without compromising effectiveness.

Furthermore, another patient characteristic that has previously been suggested to be associated with poorer antidepressant treatment response and tolerability is female sex (39). This is particularly important as MDD can often be more prevalent in females than males (61.97% versus 38.03% at baseline in the current study and 65.9% versus 34.1% at baseline in the RELEIVE China study) (3, 33). However, in this study, no significant differences were observed between males and females in either AEs or effectiveness estimates.

In patients with liver diseases, antidepressants can have a prolonged half-life and reduced drug clearance, necessitating careful monitoring of dosing, concurrent medications and other factors to reduce the risk of hepatic AEs such as GI bleeding (40). Although vortioxetine is primarily metabolized by the liver, the use of vortioxetine was safe and effective in patients with liver diseases in the current study; building on previous evidence of no clinically meaningful differences in its pharmacokinetic profile in patients with liver diseases compared to healthy counterparts (41).

Weight gain is commonly observed during the course of treatment with antidepressants, which can subsequently affect metabolic parameters in patients with MDD and/or reduce adherence to antidepressants (10, 42). In the current study, the change to the mean weight at Visit 3 (24±2 weeks) from baseline was minimal, which is in line with the known safety profile from clinical trials and other real-world studies of vortioxetine (19, 23, 36). For example, a recent meta-analysis of RCTs reported that no clinically relevant weight changes were observed in diabetic patients with MDD over time (21). Sexual dysfunction is another common AE observed in patients receiving antidepressants (43), especially in those receiving SSRIs and SNRIs (44). In this study, the occurrence of sexual dysfunction was rare (0.06% in males), suggesting vortioxetine as a potential alternative for those experiencing sexual dysfunction with other antidepressants.

This was the first long-term, nationwide PMS study of vortioxetine in patients with MDD in South Korea. Given the broad inclusion criteria of the study and the high number of included patients (3,263 in the safety assessment), the study population is likely to be representative of the real-world population of patients being prescribed vortioxetine in South Korea. Nevertheless, the study also had several limitations that should be considered. While the current study followed patients for up 6 months (24±2 weeks) which is a much longer period than RCTs (6 or 8 weeks), this 6-month follow up might not be sufficient to reflect the longer-term outcomes of patients with MDD in the real-world setting. Furthermore, real-world settings may differ from one region to another, and thus the results of this study may not be generalizable to other real-world studies. However, despite the potential differences between the real-world situation in South Korea and elsewhere, the safety and effectiveness results reported in this population showed a similar picture to previously published data.

Overall, the current study shows that the use of vortioxetine was safe and effective for the treatment of patients with MDD in a real-world setting in South Korea, which reflects the known profile of vortioxetine based on studies worldwide. Improvements in function, severity of depression and cognitive function were reported, with the treatment being safe and well tolerated. These results were also seen in elderly patients and no differences in terms of sex or liver impairment were observed. This suggests vortioxetine could be a safe and effective treatment option in the wide variety of MDD patients seen in the real-world clinical setting.

## Data availability statement

The original contributions presented in this study are included in this article/supplementary material, further inquiries can be directed to the corresponding author.

## Ethics statement

The studies involving human participants were reviewed and approved by Institutional Review Boards at each study site or a centralized Institutional Review Board, designated by Ministry of Health and Welfare. The patients/participants provided their written informed consent to participate in this study.

## Author contributions

ER and ML: substantial contributions to the study conception and design. DK, SM, JK, KL, ER, ML, and YP: substantial contributions to the analysis and interpretation of the data, drafting the article or revising it critically for important intellectual content, and final approval of the version of the article to be published. All authors contributed to the article and approved the submitted version.
